# Evidence of Concussion Signs in National Rugby League Match Play: a Video Review and Validation Study

**DOI:** 10.1186/s40798-017-0097-9

**Published:** 2017-08-22

**Authors:** Andrew J. Gardner, David R. Howell, Christopher R. Levi, Grant L. Iverson

**Affiliations:** 1 Centre for Stroke and Brain Injury, School of Medicine and Public Health, University of Newcastle, Callaghan, Australia; 2 Hunter New England Local Health District Sports Concussion Program, John Hunter Hospital, Newcastle, New South Wales Australia; 3 The Micheli Center for Sports Injury Prevention, Waltham, MA USA; 4 Division of Sports Medicine, Department of Orthopaedics, Boston Children’s Hospital, Boston, USA; 5 Brain Injury Center, Boston Children’s Hospital, Boston, MA USA; 6 Center for Health and Rehabilitation, Department of Physical Medicine and Rehabilitation, Harvard Medical School, 79/96 Thirteenth Street, Charlestown Navy Yard, Charlestown, MA USA; 7 Spaulding Rehabilitation Hospital, Charlestown, USA; 8 MassGeneral Hospital for Children™ Sport Concussion Program, and Home Base, A Red Sox Foundation and Massachusetts General Hospital Program, Boston, MA USA; 9 Priority Research Centre for Stroke and Brain Injury, Level 5, McAuley Building, Calvary Mater Hospital, Waratah, NSW 2298 Australia

**Keywords:** Concussion, Video analysis, Injury management, Return to play

## Abstract

**Background:**

Many professional sports have introduced sideline video review to help recognise concussions. The reliability and validity of identifying clinical and observable signs of concussion using video analysis has not been extensively explored. This study examined the reliability and validity of clinical signs of concussion using video analysis in the National Rugby League (NRL).

**Methods:**

All 201 professional NRL matches from the 2014 season were reviewed to document six signs of possible concussion (unresponsiveness, slow to get up, clutching/shaking head, gait ataxia, vacant stare, and seizure).

**Results:**

A total of 127,062 tackles were reviewed. Getting up slowly was the most common observable sign (2240 times in the season, 1.8% of all tackles) but only 223 times where it appeared to be a possible concussion (0.2% of all tackles and 10.0% of the times it occurred). Additionally, clutching/shaking the head occurred 361 times (on 212 occasions this sign appeared to be due to a possible concussion), gait ataxia was observed 102 times, a vacant stare was noted 98 times, unresponsiveness 52 times, and a possible seizure 4 times. On 383 occasions, one or more of the observable signs were identified and deemed associated with a possible concussion. There were 175 incidences in which a player appeared to demonstrate two or more concussion signs, and 54 incidences where a player appeared to demonstrate three or more concussion signs. A total of 60 diagnosed concussions occurred, and the concussion interchange rule was activated 167 times. Intra-rater reliability (κ = 0.65–1.00) was moderate to perfect for all six video signs; however, the inter-rater reliability was not as strong (κ = 0.22–0.76). Most of the signs had relatively low sensitivity (0.18–0.75), but high specificity (0.85–1.00).

**Conclusions:**

Using video replay, observable signs of concussion appear to be sensitive to concussion diagnoses when reviewing known injuries among professional rugby league players. When reviewing an entire season, however, certain signs occur very commonly and did not identify concussion. Thus, the implementation of video review in the NRL is challenging, but can provide a useful addition to sideline concussion identification and removal from play decisions.

## Key Points

- The addition of video review to the assessment of concussion injury events may help improve consistency in the management of players, as well as assist in diagnostic decision-making in cases where signs may be transient and resolved by the time of the medical assessment.
- The signs of concussion appear to be quite sensitive to concussion when reviewing *known injuries*; however, when reviewing an entire season, some signs occur very commonly and usually do not reflect a concussive injury.
- Most signs of concussion had high specificity but low sensitivity when examining all tackles across a sporting season.

## Background

Participation in many full contact and collision sports, such as rugby league, carries with it a risk of concussion [10]. In-game concussion diagnosis, however, remains a highly challenging task for the athletic trainer and sports medicine physician. On-field or sideline clinical assessments can be challenging due to the heterogeneous presentation of an athlete following a head impact, the non-specific nature of many of the clinical signs and symptoms of concussion [29], the absence of a reliable concussion biomarker [40], and the regularity with which some concussion signs emerge and evolve over time [31]. Recognising a potential concussion and removing an athlete from play is understood to be an important intervention for reducing the risk of a worse clinical outcome following injury [31]. However, it is acknowledged that in some instances, concussions may be missed from the sideline [25]. This may occur for a variety of reasons, but commonly the transient early physical signs may resolve before the player can be removed from play and assessed [25].

Some prior studies suggest that worse outcomes following concussion are associated with on-field signs and symptoms, such as loss of consciousness [28], amnesia [8, 28], mental status change for more than 5 min [8], and dizziness [23]. It is important to appreciate that the literature on the association between on-field signs and symptoms is mixed. For example, loss of consciousness has been associated with worse clinical outcomes in some [1, 4, 15, 28, 35, 39], but not in most studies [2, 3, 5, 8, 9, 16, 18, 22, 27, 30, 32, 36–38, 42, 43]. The vast majority of studies examining loss of consciousness base this finding on a questionnaire or interview completed with the athlete, not video review of the injury event for confirmation. Similarly, post-traumatic amnesia has been associated with worse clinical outcomes in some [15, 24, 28], but not in most studies [1–3, 8, 16, 18, 23, 27, 30, 34, 37, 38, 42]. Dizziness has been observed as an on-field symptom associated with a protracted recovery of greater than 21 days (6.34 time more likely) [23], but assessing dizziness is subjective and may or may not manifest as an objective sign (e.g., gait ataxia). Thus, video review may allow for the quantification of objective concussion signs, but not subjective symptoms.

In the sport of rugby league, the concussion incidence rates have been reported to vary widely depending on the level of competition [10]. In one study of three National Rugby League (NRL) clubs, a concussion incidence rate of 14.8 concussions per 1000 player match hours was reported [13], while a rate of 28.3 concussion per 1000 player match hours were reported from one NRL club over a 15-year (1998–2012) period [41].

The use of video footage on the sideline for reviewing a concussion has been introduced in a number of professional sports as a method to improve the recognition of a possible concussion that may be missed by on-field medical personnel [25]. Video studies have now been conducted in a variety of sports such as rugby league [11–13], rugby union [21], hockey [7], and Australian rules football [6, 25, 26]. In addition to the introduction of sideline video review, the governing body of the sport in Australia (the National Rugby League) implemented a new the “concussion interchange rule” (CIR). The CIR requires the mandatory removal of any player suspected of having sustained a concussion. The CIR allows a player to be removed from play for 15 min to be assessed by the club medical officer, including completing the Sports Concussion Assessment Tool third edition (SCAT-3). Following the assessment, if the player was not diagnosed with concussion, they are permitted to return to play without using an interchange. The incidence of use of the CIR was 24.0 (95% CI 20.7–27.9) uses of the CIR per 1000 NRL player match hours [12], and 44.9 (95% CI 38.5–52.3) uses of the CIR per 1000 National Youth Competition player match hours [13].

The primary aim of this study was to determine the rate of six objective concussion signs that occurred during the 2014 NRL season, as well as the sensitivity and the specificity of these signs to classify whether or not a diagnosed concussion resulted from the event. The secondary objective of this study was to analyse the intra- and inter-rater reliability of these signs using the video recordings of each incident that activated the CIR.

## Methods

### Participants

Participants for this study were the entire league of NRL players involved in match play during the 2014 season. Each team is comprised of a squad of approximately 25 players, and there are 16 teams in the league. There are 13 players on the field during the match.

### Procedure

This study conducted a video analysis of every game during the 2014 National Rugby League (NRL) season. The full game video analysis for all games during the season was conducted retrospectively, at the conclusion of the season by the lead author. The video review was conducted independent of any club trainer or team medical staff. The video was used to examine all on-field signs. There was no prerequisite for recording the presence of a sign (e.g., contact with the head was not required). The lead researcher obtained information on the use of the CIR (i.e., the player’s name and round in which the CIR was used). Each NRL team physician provided a list of players who had been diagnosed with concussion during the season. The club physician made the final diagnosis of concussion, based on conventional clinical examination techniques. For 162/167 (97%) uses of the CIR [12], and for all diagnosed concussions (*n* = 60), the Observational Review and Analysis of Concussion (ORAC) form [14] was used to record the game circumstances and details of the event as well as the presence or absence of six concussive signs (clutching or shaking head, slow to get up, gait ataxia, blank/vacant stare, unresponsiveness, and seizure). There were five uses of the CIR where the video of the incident was not located; none of these cases were diagnosed with a concussion. There were 162 (97%) cases where the CIR incident was identified, and 60 (100%) cases were medically diagnosed with a concussion. Two reviewers conducted the video analysis of these cases. To assess intra-rater reliability, one of the reviewers also completed a second review of 162 video clips of uses of the CIR, which included a review of all cases of medically diagnosed concussion.

All diagnosed concussions and 97% of the uses of the CIR were independently reviewed by two raters. The video raters were blinded to the club medical staff’s documentation (i.e., they conducted their retrospective review of the video signs completely independently of the club medical staff). Both raters have experience in the identification of concussion on the sideline and with retrospective video review. The two raters determined whether any of six signs of concussion were present, absent, or indeterminable based on the available footage of the incident for every case. When there was disagreement between the two raters, both raters reviewed and discussed those cases in an effort to reach consensus. In the cases where consensus could not be achieved, ratings from an experienced third rater were used. Consent was obtained from all participants who used the CIR. This study was approved by the University of Newcastle Human Ethics Committee (H-2012-0344).

The ORAC form was created to provide a simple but standardised framework for coding and analysing video footage of the situations and consequences of concussion events in rugby league. The form was developed based on work conducted previously in ice hockey in North America [19], but was adapted to include specific information to rugby league. The form includes concussion signs that have been previously examined in video review studies in rugby league [11–13]. The form consists of various sections related to the player and game characteristics (e.g., ball carrier versus tackler, tackle height, type of play, etc.), the anatomical region of contact, the injury location on the field of play, the injured player’s on-field management, and six concussive signs (defined in Table 1).

**Table 1:** Definition of video signs

Concussion sign	Definition
Clutch or shake head	The player holds his head or face in the palm of his hand or hands, or the player rubs or shakes his head in a manner that appears to demonstrate they are experiencing discomfort (i.e., not just wiping his face).
Slow to return to feet/play	Player took longer than usual to return to his feet (e.g., remained on the ground, got to his knees or to his haunches, and waited momentarily before standing), in the absence of another player holding him down.
Gait ataxia (wobbly legs)	Unable to stand steadily unaided or walk normally. Appears “clumsy”. Upon standing and walking the player has unsteadiness, wobbly legs, balance problems, stumbles or falls over, drags his feet, or cannot walk straight independently.
Blank/vacant stare	The player is not visually focused on doctor/trainer when being spoken to, or assessed and asked to attend, and/or the player appears to be looking off into the distance. The player’s face is expressionless.
Evidence of unresponsiveness	The player’s body goes limp/floppy, the player loses control of muscles, the player *does not* protect (i.e., brace) himself when falling (e.g., ragdoll-like appearance). The player remains/lies motionless on the ground for a period of time longer than expected, or the player shows a lack of visible responsiveness to verbal stimuli.
Post-impact seizure	Tonic posturing—stiffening of limbs; or clonic movements—involuntary repetitive contraction and relaxation of the muscles, could include jerky movements, or shaking/convulsion of upper and/or lower limbs, or body.

**Fig. 1:**
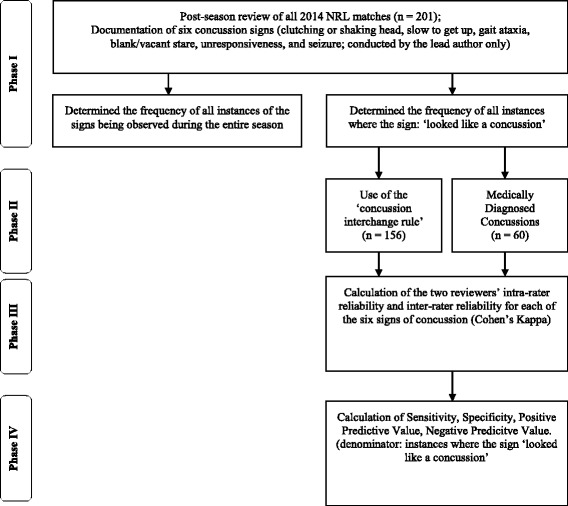
Hierarchy of methods and procedures of video review process

The full-match digital records of all 2014 NRL season matches (*n* = 210) were reviewed by a single rater (AG) using the Quicktime Multimedia Player V.7.7.5. The season prevalence of the six concussion signs were recorded by this rater. There was an important distinction made between observing a sign and instances where the sign ‘looked like it was a concussion-related’ sign, so there were two tallies made for each sign (i.e., an entire season tally of the sign regardless of the reasons for the player displaying that sign, and a season tally of the sign where the sign appeared to reflect a possible concussion). We operationally defined ‘looked like a concussion’ as all instances where there was an absence of another explanation for the sign. The digital records of events leading to the use of the CIR and medically diagnosed concussion were independently reviewed by two raters, on two separate occasions. The only circumstances that rendered a reported use of the CIR or concussion ineligible for inclusion were (i) any event where the video of the player was obstructed by other players or the match official(s) (*n* = 0), or (ii) where the incidence was unable to be located following a full-match review (*n* = 5 uses of the CIR; *n* = 0 diagnosed concussions). The ORAC form was used to code each application of the CIR and medically diagnosed concussion, in an identical methodology to previous studies in this setting [11–13]. See Fig. 1 for details.

### Statistical Analysis

Descriptive analyses are presented as means (standard deviation) for continuous variables; categorical variables are presented as numbers included (*n*) or percentage of the total. Sensitivity, specificity, positive predictive value (PPV), and negative predictive value (NPV) were calculated for each of the six concussion signs using 2 × 2 contingency tables. The number of times a player demonstrated one or more of these signs that looked like a possible concussion sign (*n* = 383) during match play for the entire season was used as the denominator for calculating sensitivity, specificity, PPV, and NPV.

Intra- and inter-rater reliability analyses using Cohen’s kappa (κ) statistics [17] were used for 162 (97%) cases where the CIR was used and for all of those cases that were medically diagnosed with concussion, to determine consistency among the two raters for each of the six individual signs. Unlike the total percent agreement, Cohen’s kappa considers the proportional agreement that could occur simply by chance. The κ coefficients are calculated by considering the proportion of rater agreement and the expected proportion [17]. Using the interpretations of κ described by McHugh [33], κ agreement was categorised as almost perfect (>.90), strong (.80–.90), moderate (.60–.79), weak (.40–.59), minimal (.21–.39), and none (0–.20). All analyses were performed using IBM SPSS Statistics V.23.0 [20] and used two-sided tests for significance at the 0.05 level, with either 90 or 95% confidence intervals (CIs).

## Results

During the 2014 season, the CIR was used on 167 occasions (97% [*n* = 162] were located and reviewed), and there were 60 medically diagnosed concussions. The incidence rate for concussion was 8.92 (95% CI = 6.96–11.43) per 1000 National Rugby League player match hours, or was approximately one concussion every 3.35 games. There were 127,062 tackles reviewed in the 201 matches, for an average of 632.1 tackles per match (Table 2). There was an important distinction made between observing a sign, and instances where the sign looked like it reflected a concussion. For example, in the 201 games reviewed, slow to get up was observed 2240 times but of those 2240 times on only 223 occasions slow to get up looked like it may have been a concussion-related sign (10.0%). Therefore, on 2017 occasions a player was slow to get up due to reasons other than a possible concussion. For example, the player stayed down to stay out of the way of play and avoid being penalised = 837, appeared to be fatigued = 706 or sustained another injury = 365; play had stopped = 78; or the opposition scored and the player stayed down = 31. The sign clutching or shaking head was observed 361 times and for 212 times the sign was considered possibly concussion-related (58.7%). Examples of when it was not considered concussion-related included when the player sustained a facial (non-nose) injury = 64 or a nose injury = 42, exhibited a disappointed or dismayed reaction to play = 27; wiped or shook off sweat from his head = 15, or appeared to have been poked in the eye = 1. All instances of observing unresponsiveness (*n* = 52), gait ataxia (*n* = 102), vacant stare (*n* = 98), and a post-impact seizure (*n* = 4) throughout the season were considered to be possibly concussion-related (Table 3).

**Table 2:** Data summary of video analysis from the 2014 National Rugby League season

Variable	Total
Matches	201
Total number of tackles in the season	127,062
Average number of tackles per match	632.1
Average concussion interchange rule user per match	0.78
Concussion interchange rule (CIR) uses	167
Returned to play	80
No return to play	76
Diagnosed concussions	60
CIR, no return to play, and no concussion	16

Note: video footage for 5 cases of the use of the CIR could not be located and therefore were not coded for concussion signs

There were 6 CIR cases where return to play data is missing

**Table 3:** Summary of the rates of concussion signs for the entire season, concussion-related signs, use of the CIR, and medically diagnosed concussions

Concussion sign	Entire season total	Possible concussion sign	Use of the CIR	CIR missing data^a^	Medically Diagnosed concussion	Medically Diagnosed concussion missing data
			(*n* = 162)^a^		(*n* = 60)	
Unresponsiveness	52	52	50	2	24	1
Slow to get up	2240	223	153	0	60	0
Clutching head	361	212	110	1	38	0
Gait ataxia	102	102	87	18	35	11
Vacant stare^b^	98	98	93	18	45	6
Possible seizure	4	4	3	0	3	0

Note: *CIR* concussion interchange rule

The “Entire Season Total” refers to every instance during the season that the sign was observed (whether it appeared to be a sign of concussion or not). The “Possible Concussion Sign” refers to the subset of the Entire Season Total in those instances where the sign was observed, and the sign appeared to be a possible concussion

^a^Video footage for 5 cases of the use of the CIR could not be located and therefore were not coded for concussion signs

^b^ The frequency of missing data for the sign ‘vacant stare’ for the entire season total was not recorded

Of the 223 instances of a player being slow to get up, 153 players were removed under the CIR (68.6%); return to play data was available for only 156/162 (96.3%) instances of the use of the CIR. Of those 153, 60 were medically diagnosed as having a concussion (39.2%), and all players who were medically diagnosed with concussion demonstrated the sign “slow to get up”. The PPV and NPV for slow to get up were 0.27 and 1.00, respectively. Of the 212 instances of clutching or shaking head, 110 players were removed under the CIR (51.9%) and 38 were diagnosed with a concussion (34.5%). Of those who were diagnosed with concussion, players clutched or shook their heads 63.3% (38/60) of the time. The PPV and NPV for clutching or shaking head were 0.18 and 0.87, respectively. Of the 102 instances of gait ataxia, 87 players were removed under the CIR (85.3%) and 35 were diagnosed with concussion (40.2%). Gait ataxia was seen in 58.3% of players medically diagnosed with concussion. The PPV and NPV for gait ataxia were 0.34 and 0.91, respectively. Of the 98 instances of a blank or vacant stare, 93 players were removed under the CIR (94.9%) and 45 were diagnosed with concussion (48.4%). A blank or vacant stare was observed in 75.0% of players medically diagnosed with concussion. The PPV and NPV for blank or vacant stare were 0.46 and 0.95, respectively. Of the 52 instances in which a player appeared to have brief unresponsiveness following a tackle, 50 players were removed under the CIR (96.2%), of which 24 were diagnosed with a concussion (48%). Unresponsiveness on the field was observed in 40.0% (24/60) of players medically diagnosed with concussion. The PPV and NPV for unresponsiveness were 0.46 and 0.89, respectively. Of the 4 instances in which a player appeared to experience a post-impact seizure, 3 players were removed under the CIR (75.0%). All three players were medically diagnosed with a concussion (100%). There were three (5%) players who were medically diagnosed with concussion that showed signs of seizure-like activity. The PPV and NPV for seizure-like activity were 0.75 and 0.85, respectively (Table 4).

**Table 4:** Rates, sensitivity, specificity, positive predictive value, and negative predictive value for concussion signs

Concussion sign	Sign present	Medically Dx concussion with sign	Sign absent	Medically Dx concussion without sign	Sign sens	95% CI	Sign spec	95% CI	PPV	95% CI	NPV	95% CI
Clutching or shaking head	212	38	171	22	0.63	0.51–0.75	0.46	0.44–0.48	0.18	0.14–0.21	0.87	0.83–0.91
Slow to get up	223	60	160	0	1.00	0.93–1.00	0.50	0.48–0.50	0.27	0.25–0.27	1.00	0.97–1.00
Gait ataxia	102	35	281	25	0.58	0.46–0.70	0.79	0.77–0.81	0.34	0.27–0.41	0.91	0.86–0.94
Blank or vacant stare	98	45	285	15	0.75	0.63–0.84	0.84	0.81–0.85	0.46	0.39–0.52	0.95	0.92–0.97
Unresponsiveness	52	24	331	36	0.40	0.29–0.50	0.91	0.89–0.93	0.46	0.34–0.58	0.89	0.87–0.91
Possible seizure	4	3	379	57	0.05	0.02–0.07	1.00	0.99–1.00	0.75	0.22–0.99	0.85	0.84–0.85

Note: All calculations were made using the number of times the sign was seen in concussed players, contrasted to the total number of times one or more signs (that appeared to represent a concussion) were seen (*n* = 383), for the entire season

≥ greater than or equal to, *CI* confidence interval, *Dx* diagnosed, *n* number, *NPV* negative predictive value, *PPV* positive predictive value, *Sens* sensitivity, *Spec* specificity

One or more video signs of possible concussion were seen during 383 tackles (i.e., only 0.3% of the total number of tackles), most commonly demonstrating the characteristic “slow to get up” (Table 5). Considering five signs simultaneously (unresponsiveness, clutching/shaking head, gait ataxia, slow to get up, or blank/vacant stare), 34 (57%) of players medically diagnosed with concussion had three or more observable signs of concussion. There were a greater total number of signs observed in the concussed group compared to players that used the CIR and were cleared to return to play in the same game (Table 5). For the individual signs, those diagnosed with concussion were more likely to show evidence of unresponsiveness and a vacant stare. There was no difference between the number of observed signs between groups for clutching or shaking the head, or gait ataxia.

**Table 5:** Summary of video analysis findings for those situations in which the concussion interchange rule was used

Signs of concussion	Medically diagnosed concussion (*n* = 60)			No diagnosed concussion and no return to play^a^ (*n* = 16)			Returned to play^a^ (*n* = 80)			Diagnosed concussion vs. returned to play^a^		
	Present	Absent	Missing	Present	Absent	Missing	Present	Absent	Missing			
	% (*n*)	% (*n*)	% (*n*)	% (*n*)	% (*n*)	% (*n*)	% (*n*)	% (*n*)	% (*n*)	χ^2^	*p*	RR (90% CI)
Unresponsiveness	40 (24)	58.3 (35)	1.7 (1)	43.8 (7)	56.3 (9)	0 (0)	23.8 (19)	75 (60)	1.3 (1)	4.35	.037	1.52 (1.06–2.10)
Slow to get up	100 (60)	0 (0)	0 (0)	100 (16)	0 (0)	0 (0)	96.3 (77)	3.7 (3)	0 (0)	Not applicable		
Clutching head	63.3 (38)	36.7 (22)	0 (0)	87.5 (14)	12.5 (2)	0 (0)	72.5 (58)	26.3 (21)	1.3 (1)	1.62	.203	0.77 (0.55–1.12)
Gait ataxia	58.3 (35)	23.3 (14)	18.3 (11)	62.5 (10)	37.5 (6)	0 (0)	52.5 (42)	38.8 (31)	8.8 (7)	2.43	.119	1.46 (0.94–2.38)
Vacant stare	75 (45)	15 (9)	10 (6)	62.5 (10)	37.5 (6)	0 (0)	47.5 (38)	37.5 (30)	15 (12)	10.43	.001	2.35 (1.40–4.30)
Possible seizure	5 (3)	95 (57)	0 (0)	0 (0)	100.0 (16)	0 (0)	0 (0)	100 (80)	0 (0)	Not applicable		
Total signs observed (*n*)	205	–	–	64	–	–	174	–	–	–	–	–

Note. Video footage for 5 cases of the use of the CIR could not be located and therefore were not coded for concussion signs; there were 6 CIR cases where return to play data is missing

^a^Return to play refers to return to play in the same game; *RR* risk ratio

The players who were medically diagnosed with a concussion were significantly more likely (1.52 times) to exhibit unresponsiveness and a vacant stare (2.35 times) than players who were removed from play under the CIR, not diagnosed with a concussion, and were returned to play. Clutching the head and gait ataxia were not significantly different signs between those who were and were not medically diagnosed with a concussion (see Table 1).

A sign that was always present in cases of diagnosed concussion was slow to get up (sensitivity = 100%), although it had low specificity (50%). A possible seizure was observed only four times during the season and on three occasions those athletes were medically diagnosed as having a concussion (3/60 diagnosed concussions; sensitivity = 5%, specificity = 100%). A blank or vacant stare had fairly high sensitivity and specificity (75% and 84%, respectively; see Table 4).

The intra-rater reliability of the video analysis of each sign of concussion was moderate to perfect, and perfect agreement was observed in three (clutch or shake head, slow to get up, and post-impact seizure) of the six signs. Moderate inter-rater reliability was observed in three of six of signs: clutch or shake head, slow to get up, and unresponsiveness (Table 6).

**Table 6:** Intra-rater reliability and inter-rater reliability for the concussion signs

	Intra-rater (κ) (95% CIs)	McHugh [33] κ agreement classification	Inter-rater (κ) (95% CIs)	McHugh [33] κ agreement classification	Absolute disagreement (CIR cases, *n* = 162)
Clutch of shake head	1.00 (–,–)	Perfect	0.76 (0.60–0.91)	Moderate	43
Slow to get up	1.00 (–,–)	Perfect	0.64 (0.42–0.83)	Moderate	27
Gait ataxia	0.77 (0.63–0.90)	Moderate	0.37 (0.21–0.53)	Minimal	36
Blank/vacant stare	0.65 (0.44–0.84)	Moderate	0.22 (0.05–0.40)	Minimal	71
Unresponsiveness	0.84 (0.70–0.94)	Strong	0.75 (0.56–0.92)	Moderate	22
Post-impact seizure	1.00 (–,–)	Perfect	0.40 (0.00–0.72)	Weak	3

Note. *CIs* confidence intervals, *κ* Kappa McHugh, κ agreement was categorised as almost perfect (>.90), strong (.80–.90), moderate (.60–.79), weak (.40–.59), minimal (.21–.39), and none (0–.20)

## Discussion

To expand previous video analysis work in collision sports, this study explored the rate of six observable signs of concussion, as well as their sensitivity and specificity during match play of a National Rugby League season. The results of the current study revealed moderate to perfect intra-rater reliability or better for all video signs. For inter-rater reliability, clutching or shaking head, slow to get up, and unresponsiveness had moderate inter-rater reliability, whereas post-impact seizure had weak inter-rater reliability, and gait ataxia and blank or vacant stare had poor inter-rater reliability. These findings are relatively consistent with those reported from a video review study of concussion signs during Australia Football League (AFL) match play, which found that intra-rater reliability was generally better than inter-rater reliability, and blank or vacant stare was the sign with the lowest agreement [25].

Blank or vacant stare was the only sign that had reasonably high sensitivity (75%) and specificity (84%); all other signs did not have both high sensitivity and specificity for a concussion diagnosis. Some of the signs had high sensitivity but low specificity, suggesting that those signs may be a useful marker in flagging a potential concussion, but not helpful in confirming the diagnosis. Therefore, medical personnel working in tandem on the sideline and with individuals reviewing video may be an appropriate strategy to achieve a sensitive and specific approach to concussion diagnosis. In a video review of concussion signs during match play in the Australian Football League (AFL), slow to get up had the highest sensitivity (87%), but low specificity (19%). All other video concussion signs examined in this prior study had high specificity but low sensitivity, such as blank and vacant look [specificity 100%, sensitivity 9%], motor incoordination [specificity 95%, sensitivity 29%], impact seizure [specificity 93%, sensitivity 7%], and rag doll appearance [specificity 91%, sensitivity 16%]). The highest PPV was found with the “blank and vacant look” (100%), and “motor incoordination” (81%) signs [25]. However, in the current study, we found that the highest PPV was for seizure (75%), while slow to get up (100%), blank or vacant stare (95%), and gait ataxia (91%) were the signs with the highest NPV.

The sign with the highest relative risk of diagnosed concussion was a blank or vacant stare (2.35 times more likely; 90% CI = 1.40–4.30), while unresponsiveness was 1.52 times more likely (90% CI = 1.06–2.10). Gait ataxia (1.46 times more likely; 90% CI = 0.94–2.38) and clutching or shaking head (0.77 times more likely; 90% CI = 0.55–1.12) were not statistically different. Blank or vacant stare appears to be a conceptually different sign to the other five. The detection of a blank or vacant stare tends to be through a secondary screening process, in that it is often only observed if, and when, a player is injured and the camera zooms in closely. It is also arguably the most subjective of the six signs and therefore vulnerable to greater variation between raters when classifying this sign. This notion is supported by the weaker inter-rater reliability of this sign not only in the current study but also in previous video review studies [12, 13, 25]. In our study, the low inter-rater reliability was due to one rater seeing the sign more often than the other, recording it as present, and then both raters ultimately agreeing that it was present. Observing a blank or vacant stare was rare in a recently published NHL video review study [7]. We do not know why the sign was so uncommon in that study compared to ours. It might be more difficult to see the sign through a hockey helmet, and it might also be more difficult to see it based on camera angles when players are moving off the ice. Among the other signs, inter-rater reliability was the strongest for clutch or shake head, slow to get up, and unresponsiveness, each with a moderate agreement classification. These signs may be more easily seen during field-side evaluations; their intra-rater reliability was strong to perfect. In contrast, the minimal agreement between raters for gait ataxia or blank/vacant stare warrants caution with their use.

There were several limitations of the current study. In some instances, the available video footage was not sufficiently clear to code all signs (i.e., the view from the available camera angle was obscured, or a close up of the incident was not available). This ‘missing data’ was excluded from the analyses, which might have slightly improved the support for the utility of some of the visible signs. This was particularly true for the blank or vacant stare sign, where missing data on this sign was common. Despite being the most sensitive and specific sign for concussion diagnosis, blank or vacant stare had the worst inter-rater reliability (0.22, with 71 absolute disagreements between raters). For these reasons, if video review within professional sports are implemented, then access to high quality reviews with the capability of multi-angle and slow motion replays to allow for close ups would be optimal [25] and would reduce the likelihood of missing data. Although the video reviewer was blinded to the sideline assessment results and the medical diagnosis of concussion for this study, they were only partially blinded to the use of the CIR. Given that the process for enacting a use of this rule requires the trainer to provide a signal to the sideline, and the official on the sideline identifies the interchange with a green card, the video reviewer was able to identify many instances where the CIR was used. Another study limitation was that there were no concussions that were subsequently (beyond match day) diagnosed by the club medical and reported to the researchers, which is inconsistent with other video review studies that have reported a numbers of cases of post-game diagnosis of concussion [7]. A further limitation of the current study pertains to the generalizability of the current findings to other levels of rugby league. The current study was a post-game review of an adult, male, professional league, and as such the results may not necessarily be generalizable to in-match real-time analysis, or other levels of match play. Our use of an operational definition of exclusion to categorise the observed signs into ‘plausible concussion signs’ versus signs that were more likely attributable to other factors, was also a limitation in terms of its subjectivity and reproducibility for future work in video review of concussion signs. Finally, only one reviewer completed the coding of the entire game, for every game in the season; the inter-rater reliability of that type of coding is unknown.

## Conclusions

A conservative approach to sideline concussion management would be to remove a player from play based on any evidence of possible concussion signs. This approach is encouraged as the best management strategy and in the best interest of the welfare of the player [31]. However, there is limited information available on the reliability and validity of identifying the objective signs of concussion when using video analysis, and indeed not all instances of observed concussion signs occur as a result of the player having sustained a concussion. The signs of concussion appear to be quite sensitive to concussion when reviewing known (i.e., medically diagnosed concussions) or suspected (i.e., players who have used the CIR) injury. When reviewing an entire season of match play, specific objective concussion signs such as slowness in getting up and clutching/shaking the head occurred commonly during professional rugby league match play, but did not typically reflect concussion occurrence. For these reasons, video injury surveillance can be difficult to interpret, but may provide a useful adjunct to the clinical assessment of potential concussion. With improved access to video replays, clear definitions and education regarding the observable signs, and improved communication between video observers and sideline medical personnel, the detection of concussion may improve.
